# Seismological evidence for a localized mushy zone at the Earth’s inner core boundary

**DOI:** 10.1038/s41467-017-00229-9

**Published:** 2017-08-01

**Authors:** Dongdong Tian, Lianxing Wen

**Affiliations:** 1 Laboratory of Seismology and Physics of Earth’s Interior; School of Earth and Space Sciences, University of Science and Technology of China, Hefei, Anhui 230026 China; 2 Department of Geosciences, State University of New York at Stony Brook, Stony Brook, New York 11794 USA

## Abstract

Although existence of a mushy zone in the Earth’s inner core has been hypothesized several decades ago, no seismic evidence has ever been reported. Based on waveform modeling of seismic compressional waves that are reflected off the Earth’s inner core boundary, here we present seismic evidence for a localized 4–8 km thick zone across the inner core boundary beneath southwest Okhotsk Sea with seismic properties intermediate between those of the inner and outer core and of a mushy zone. Such a localized mushy zone is found to be surrounded by a sharp inner core boundary nearby. These seismic results suggest that, in the current thermo-compositional state of the Earth’s core, the outer core composition is close to eutectic in most regions resulting in a sharp inner core boundary, but deviation from the eutectic composition exists in some localized regions resulting in a mushy zone with a thickness of 4–8 km.

## Abstract (web summary)

The existence of a mushy zone in the Earth’s inner core has been suggested, but has remained unproven. Here, the authors have discovered a 4–8 km thick mushy zone at the inner core boundary beneath the Okhotsk Sea, indicating that there may be more localized mushy zones at the inner core boundary.

## Introduction

The thermo-compositional state of the Earth’s core is generically related to the growing process of the inner core and its associated releases of the thermal and compositional energy that power the Earth’s geodynamo^1–4^. It has long been proposed that the thermo-compositional state of the Earth’s core is such that the interface between the inner and outer core is dendritic with the mushy zone possibly even extending throughout the entire inner core^5^. However, while recent studies have revealed various degrees of complexicity near the inner core boundary (ICB), such as hemispherical variations of seismic properties at the top of the inner core^6–10^, laterally varying seismic properties near the inner core surface^11–13^ and spatially varying ICB topography^14–18^, no seismic evidence of a mushy zone has ever been reported.

Here we present seismic evidence for a localized 4–8 km thick mushy zone across the ICB beneath southwest Okhotsk Sea, with seismic properties intermediate between those of the inner and outer core. Such a mushy zone is also studied in the context of its surrounding ICB regions, which are found to have a sharp boundary. These seismic results suggest that, in the current thermo-compositional state of the Earth’s core, the outer core composition is close to eutectic in most regions resulting in a sharp ICB, but deviation from the eutectic composition exists in some localized regions resulting in a mushy zone with a thickness of 4–8 km.

## Results

### Approach to study the inner core boundary

**Fig. 1:**
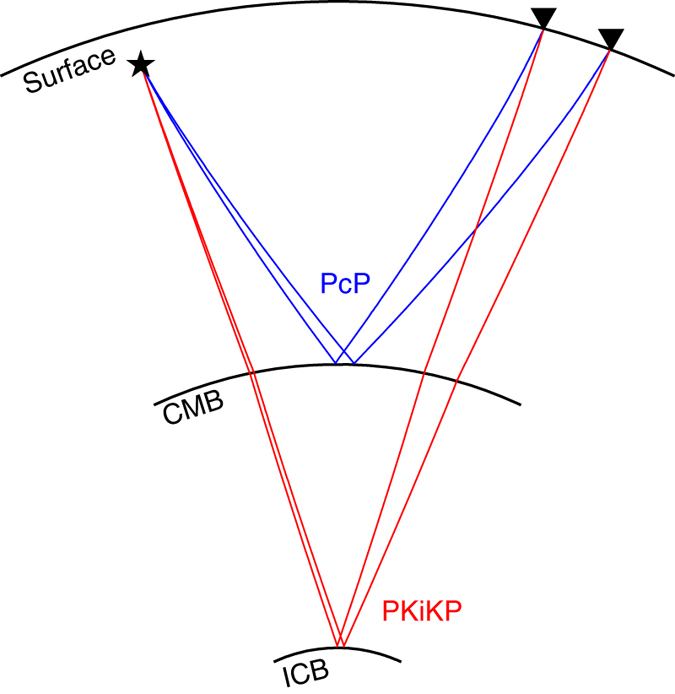
Raypaths of PKiKP and PcP phases. PKiKP (*red*) and PcP (*blue*) are compressional waves reflected off the inner core boundary (*ICB*) and the core–mantle boundary (*CMB*) respectively. Raypaths are calculated based on PREM^29^, for a seismic source at a depth of 300 km (*black star*) and receivers (*black triangles*) at two example epicentral distances of 30° and 35°

Our study is based on the analyses of differential travel times and waveforms between seismic PKiKP and PcP phases, compressional waves that are reflected off the Earth’s ICB and the core–mantle boundary (CMB) respectively (Fig. 1), recorded in the epicentral distance range of 0–90°. PKiKP–PcP differential travel time residuals are used to study the topographic variations of the ICB, while the waveform differences between the two phases are used to explore the detailed features of the ICB. Owing to the similar raypaths between PKiKP and PcP phases in the shallow Earth (Fig. 1), the use of PKiKP–PcP differential travel time residuals eliminates the uncertainty of event origin time and minimizes the errors of event mislocation and the effects of shallow Earth’s heterogeneities, placing high-resolution constraints on the ICB topography. For the similar reasons, because of the similar take-off angles from the seismic source and similar incident angles to the receiver between PKiKP and PcP waves, the study of their waveform differences eliminates possible effects on waveform complexities due to source complication and heterogeneous seismic structure beneath the receivers, providing tight constraints on the fine-scale seismic structure in the vicinity of the ICB.

### Seismic data and regions of inner core boundary studied

**Fig. 2:**
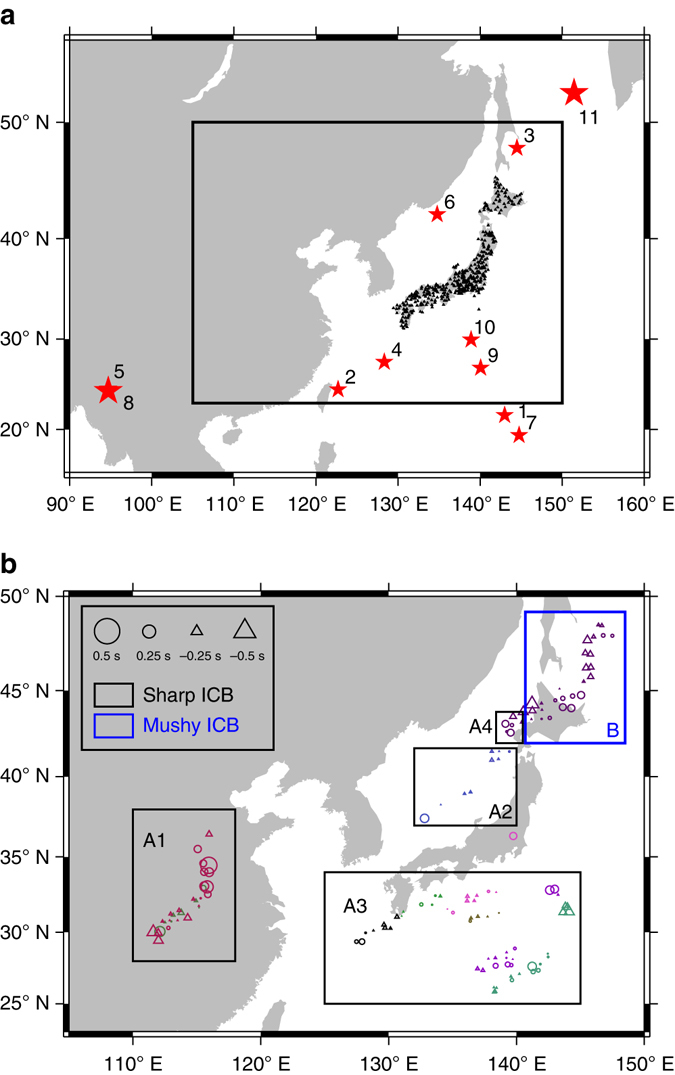
ICB study region and PKiKP–PcP differential travel time residuals. **a** Map view of the ICB study region (*solid black box*, with detailed results shown in Fig. 2b), along with seismic events (*red stars*, labeled by event ID listed in Supplementary Table 1) and stations (*black triangles*) used in the analyses. **b** Geographic distribution of PKiKP–PcP differential travel time residuals in the study region (corresponding to the *solid black box* in Fig. 2a), averaged over caps with a radius of 1° covering the PKiKP reflected points at the ICB (Supplementary Fig. 1 for a complete version). Cap-averaged residuals are plotted at the center of each cap and color-coded with each event, with positive and negative values denoted by *circles* and *triangles* respectively and the size of the symbol proportional to the absolute value of travel time residual. The ICB is grouped into regions according to their structural characteristics inferred based on seismic data, with *black boxes* (labeled as A: A1, A2, A3, and A4) indicating flat and sharp regions and *blue box* (labeled as B) a region with a laterally varying double-layered structure (mushy zone) across the ICB

Our study region lies between 25–50° N in latitude and 110–150° E in longitude (Fig. 2a), sampled by the seismic data recorded in a dense seismic array in Japan, Hi-net^19^. We select all the events recorded by Hi-net from April 2004 to December 2014 that satisfy following three criteria: epicentral distances to the Hi-net stations are from 0° to 90° in which PKiKP phase is pre-critically reflected from the ICB and is most sensitive to the detailed seismic structure at the top of the inner core^20–27^, focal depths are greater than 30 km to avoid the contamination of crustal wave reverberations near earthquake sources, and earthquake magnitudes are greater than Mw 5.8, large enough to generate observable PKiKP phases. Such criteria yield a total of 1106 events. With the inclusion of an additional 2001 event (event 1, Fig. 2a) that was used in a previous ICB study^28^, our data set consists of over 850,000 potential PKiKP–PcP pairs. After eye-checking data quality of each seismogram, we are able to retain a total of 1263 pairs of high-quality PKiKP and PcP waveforms from 11 seismic events (Fig. 2a, Supplementary Table 1). These seismic data sample the ICB regions beneath central China, southeast Japan Sea, west North Pacific Ocean, and southwest Okhotsk Sea (Fig. 2b, Supplementary Fig. 1).

### Non-significant inner core topography in the study region

In the travel time analysis of ICB topography, we remove instrumental responses from waveform data and then apply the worldwide standard seismic network short-period instrumental response and a two-pole causal Butterworth bandpass filter of 1–3 Hz. Such filtering is the procedure we find to have the best observability of PKiKP phases in the Hi-net data after extensive tries of various procedures of frequency filtering. We then measure the PKiKP–PcP differential travel times based on the time separation of the maximal amplitudes of the two phases, and calculate PKiKP–PcP differential travel time residuals with respect to the predictions of a seismic reference model, PREM^29^ (see “Obtaining PKiKP–PcP differential travel time residuals” in Methods).

Detailed data analyses confirm that using PKiKP–PcP differential travel time residuals significantly minimizes the effects of shallow mantle structure (Supplementary Fig. 2). PKiKP–PcP differential travel time residuals exhibit small variations from −0.5 to 0.5 s (with 93% of the data within −0.3 to 0.3 s) for all the data we analyze (Fig. 2b, Supplementary Figs. 2–4). If we attribute all the observed variations of PKiKP–PcP differential travel time residuals to ICB topographic variations, the estimated upper bound of ICB topographic changes in the study region is 1.5 km, similar to previous results obtained beneath the western Pacific^17^. Note that PKiKP–PcP travel time residuals exhibit slight negative correlations with PcP travel time residuals for events 2, 5, 6, 8, 10, and 11 (Supplementary Figs. 2 and 4), indicating that some of the PKiKP–PcP travel time residuals are still to some extent affected by the heterogeneities in the mantle along the PcP raypaths and the actual ICB topography could be even smaller. We conclude that there is no significant ICB topography in the study region.

### A double-layered boundary beneath southwest Okhotsk Sea

**Fig. 3:**
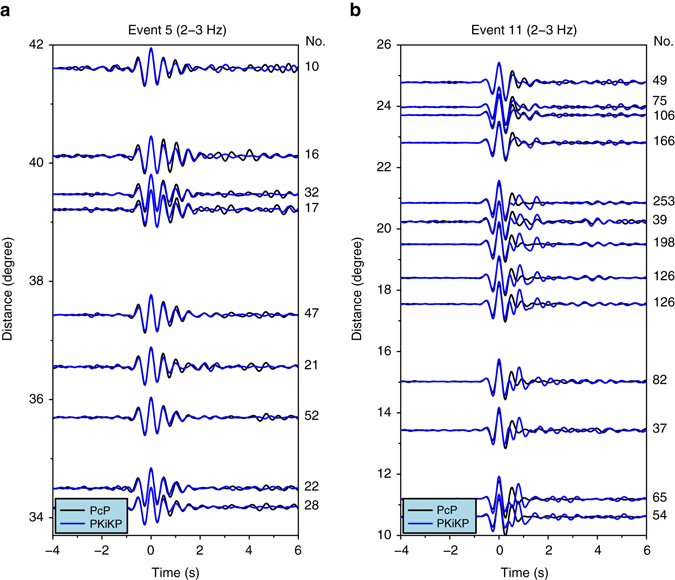
Comparisons of stacked PKiKP and PcP waveforms of two example events sampling two regions of ICB with different seismic characteristics. **a** Comparisons for event 5 which samples a sharp ICB beneath central China (*black box* labeled as A1 in Fig. 2b). **b** Comparisons for event 11 which samples a double-layered mushy ICB beneath southwest Okhotsk Sea (*black box* A4 and *blue box* B in Fig. 2b). Stacked PcP (*black traces*) and PKiKP (*blue traces*) waveforms are bandpass filtered in a frequency range of 2–3 Hz. The number of waveforms used in each stacking is indicated at the *right* of each trace

We compare stacked PKiKP and PcP waveforms for the seismic data sampling various regions of the ICB (see “Waveform stacking” in Methods). The stacked PKiKP and PcP waveforms are similar for most events, including those sampling the ICB regions beneath central China, southeast Japan Sea and west North Pacific Ocean (Fig. 3a for an example event and Supplementary Fig. 5 for other events) and some portion of the ICB region beneath southwest Okhotsk Sea (Fig. 3b in the distance range larger than 24°). Waveform modeling indicates that a sharp ICB, no thicker than 1 km, is required in those regions so that PKiKP waveforms would maintain similarities with the corresponding PcP waveforms.

However, significant waveform differences are observed between most of the PKiKP and PcP phases for event 11 (Fig. 3b), which sample the ICB region beneath southwest Okhotsk Sea (*blue box* B in Fig. 2b). In the frequency band of 2–3 Hz, the stacked PKiKP waveforms (with a stacking radius of 2°) exhibit two pulses in the epicentral distance range from 10° to 24°, while the stacked PcP waveforms show a simple pulse (Fig. 3b). The two energy pulses in the PKiKP waveforms have a time separation of about 0.9 s, with the relative amplitudes of the second pulses decreasing gradually with increasing epicentral distances (Fig. 3b). We perform following statistical tests and these tests confirm the robustness of the stacked results. We stack PKiKP waveforms using a smaller stacking radius of 1° and the stacked waveforms exhibit the same feature of double pluses (Supplementary Fig. 6); we stack waveforms according to propagational azimuths of the seismic waves and the stacked waveforms exhibit the same feature of double pulses with no azimuthal dependency; and we apply a bootstrap resampling method^30^ to estimate the uncertainties of the stacked waveforms and double peaks stand out well above the 95% confidence intervals of the stacked PKiKP waveforms (Supplementary Fig. 7). The observed distinct double pulses in PKiKP waveforms are markedly different from the PKiKP coda waves studied in previous studies^31–33^, which have characteristics of growing energy with time after direct PKiKP arrivals for a few hundred seconds without impulsive onsets.

Several lines of evidence suggest that the anomalous PKiKP waveforms are caused by the seismic structure near the ICB, as other possible sources can be excluded. The observed anomalous PKiKP waveforms cannot be caused by a complex source or near source heterogeneities. Note that the PKiKP waveforms change from a simple single pulse at the distance of 25° to double pulses at 24° (Fig. 3b). The take-off angles from the source for the PKiKP waves recorded at these two distances have a difference of only 0.13°. A complex source would produce same waveform complexities for PKiKP waves recorded at these distances. Similarly, the separation of the two seismic paths is only 0.47 km at 800-km depth, making near source heterogeneities impossible to produce such waveform change between these two epicentral distances. They cannot be caused by the subducted slab beneath Japan. If a seismic heterogeneity exists within the slab and generates a secondary phase to the PKiKP waves of event 11, such a seismic heterogeneity would have also geographically been sampled by the PcP waves of event 11 (Fig. 3b) and the PKiKP and PcP waves of other events. However, no such secondary phases are observed for the PcP waves of event 11 and PKiKP and PcP waves of other events. Theoretical calculations also indicate that a low-velocity channel even with a compressional velocity reduction of 10% would produce a secondary phase having no more than 7% of the amplitude of the main phase, unable to account for the observations. They cannot be caused by the seismic structure at the PKiKP exit regions at the CMB. Note that the reflected points of PcP waves partially overlap with CMB exit points of PKiKP waves (*black dashed box* in Supplementary Fig. 8) and those PcP phases exhibit simple waveforms, indicating that the CMB region near the PKiKP exit points has a simple seismic structure. They cannot be caused by the seismic structure at the PKiKP entrant regions at the CMB, as this portion of the CMB region (*red triangles* in Supplementary Fig. 8) exhibits undetectable seismic scattering based on the study of PKP precursors observed in Japan islands for earthquakes that occurred in South America^34^. Theoretical analysis further indicates that the observed secondary pulses in the PKiKP waveforms cannot be caused by a low-velocity zone above the CMB either in the entrant or exit points of the PKiKP waves. Synthetic calculations indicate that, for any possible low-velocity zones above the CMB that would produce a 0.9 s delayed secondary pulse after the main PKiKP phase, the predicted amplitude of the secondary phase is <7% of that of the main phase because of the small reflection coefficients of the compressional waves off the CMB and the top of the low-velocity zone. Such small secondary pulse would not be discernable in the data and is unable to account for the strong secondary pulses observed in the PKiKP waveforms (Fig. 3b). Based on above observations and analyses, we conclude that the anomalous PKiKP waveforms are caused by seismic structure near the ICB.

**Fig. 4:**
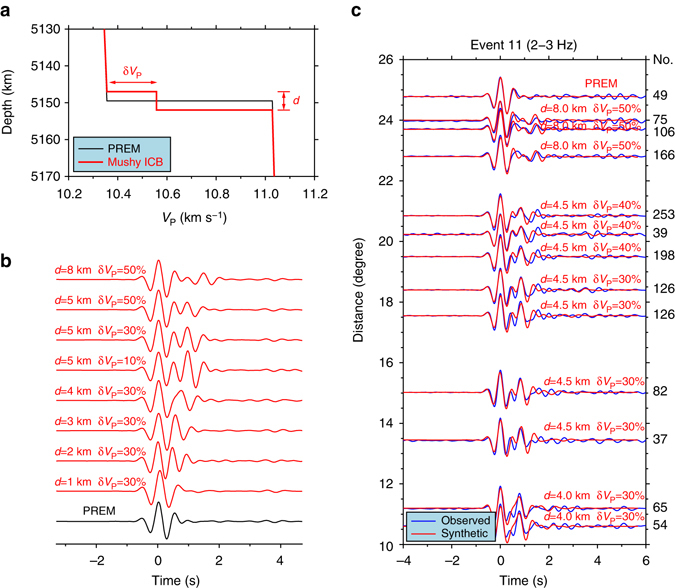
Seismic model and waveform modeling for the PKiKP data sampling the ICB region beneath southwest Okhotsk Sea. **a** Compressional velocity profile of the best-fitting double-layered model across the ICB (*red line*) represented by two parameters: *d* the thickness of the layer and δ*V*_P_ percentage compressional velocity jump of the top layer with respect to PREM velocity jump at ICB, along with PREM (*black line*). **b** Synthetic seismograms in a frequency range of 2–3 Hz for a series of double-layered ICB models (*red traces*, labeled accordingly with two model parameters: *d* and δ*V*_P_) and for PREM (*bottom black trace*). **c** Comparisons of stacked observed PKiKP waveforms (*blue traces*) of event 11 in a frequency range of 2–3 Hz and synthetic seismograms (*red traces*) of the best-fitting models. The number of waveforms used in each stacking is labeled at the *right* of each trace, while the thickness *d* and percentage compressional velocity jump of the top layer δ*V*_P_ of the best-fitting models are labeled above the *red traces*. The sampled ICB region also corresponds to Boxes B and A4 in Fig. 2b

The observed PKiKP waveforms can be explained by a double-layered ICB model with laterally varying thicknesses and compressional velocity jumps. We test double-layered ICB models with thicknesses from 1 to 10 km (with an interval of 1 km) and compressional velocity jumps from 10% to 90% (relative to values of PREM^29^, with an interval of 10%) (Fig. 4a and b). A double-layered ICB model with a thickness of 4–8 km and laterally varying compressional velocity jumps of ~30% to ~50% can well explain the observed PKiKP waveforms in the distance range of 10–24° (Fig. 4c). Note that synthetics of the best-fitting models exhibit two energy pulses that fit the observations well, in both relative timing and amplitude (Fig. 4c).

## Discussion

The double-layered structure fits exactly the characteristics of a mushy zone. Fearn et al.^5^ proposed that the mushy zone should exist in the inner core condition and the inner core solidification system should consist of three regions: a lower region of complete solid, an upper region of complete liquid and a middle region, the mushy zone, in which both solid (in dendrites) and liquid are present. We thus infer that the 4–8 km thick layer with seismic properties between the outer and inner core is a mushy zone proposed by Fearn et al.^5^.

We further note that the double-layered structure is only found in a localized region with other ICB regions characterized by a sharp boundary. Such results could be explained by a laterally varying solidification process of the inner core due to lateral variation of composition in the bottom of the outer core. In the thermo-compositional state of a mushy zone, the top of the mushy zone is controlled by the liquidus condition of the outer core composition, while the bottom of the mushy zone by the eutectic condition of the system. The thickness of the mushy zone would thus depend on the difference between the outer core composition and the eutectic composition. The sharp feature of the ICB observed in other regions can be explained by the solidification in the condition of eutectic composition, in which the mushy zone disappears. Based on our seismic results and the study of Fearn et al.^5^, we suggest that, in the current thermo-compositional state of the Earth’s core, the outer core composition is close to eutectic in most regions resulting in a sharp ICB; however, in some localized regions, deviation from the eutectic composition exists resulting in a mushy zone with a thickness of 4–8 km.

## Methods

### Obtaining PKiKP–PcP differential travel time residuals

PKiKP–PcP differential travel time residuals are defined as:

1 $${\rm{d}}T = \left( {{\rm{PKiK}}{{\rm{P}}_{{\rm{obs}}}}{\rm{-PKiK}}{{\rm{P}}_{{\rm{pre}}}}} \right){\rm{-}}\left( {{\rm{Pc}}{{\rm{P}}_{{\rm{obs}}}}{\rm{-Pc}}{{\rm{P}}_{{\rm{pre}}}}} \right)$$

where the subscripts “obs” and “pre” denote observed travel time and predicted travel time calculated based on a reference model, respectively. We measure PKiKP and PcP travel times based on the maximal amplitudes of the two phases, since the onsets of the two phases are difficult to be identified. For each event, we stack the PKiKP waveforms along the handpicked arrival times, re-pick the PKiKP arrival time in each trace based on its cross-correlation with the stacked PKiKP waveform and eye-check the waveforms in the final picking to avoid possible cycle skipping. Such picking procedure ensures the reliability and accuracy of phase picks. We apply the same procedure to measure the arrival times of PcP phases. We calculate PKiKP–PcP differential travel time residuals with respect to PREM^29^. For each event, we remove the mean value of the PKiKP–PcP differential travel time residuals from the observations.

The PKiKP travel time residuals exhibit a linear relationship with the PcP travel time residuals (Supplementary Figs. 2 and 4), indicating that most of the travel time residuals are contributed by the seismic structure of shallow Earth. The PKiKP–PcP differential travel time residuals exhibit no significant correlation with either the PcP or PKiKP travel time residuals, indicating that the effects of shallow Earth’s structure have been effectively removed from the differential PKiKP–PcP travel time residuals.

### Waveform stacking

Waveform stacking is an effective method to reduce the effects of small-scale scattering in the upper mantle and to enhance signal-to-noise ratios of the data. We first divide the PKiKP reflected regions into overlapping circular grids with a radius of *R*. We then collect traces whose PKiKP reflected points fall into each grid, self-normalize the waveforms and linearly stack the waveforms along the handpicked arrival times. Stacked waveforms are considered to be reliable only when the number of records used in the stacking exceeds a certain threshold *N*. In the present study, we choose *N* = 10 and *R* = 1° or *R* = 2°. These numbers are determined empirically to best balance reliability and spatial resolution of stacked waveforms. We apply the same procedure to stack PcP waveforms.

### Waveform modeling

We calculate synthetic seismograms by a two-dimensional hybrid method^35^, which employs a numerical finite-difference (FD) method in a localized region and analytical methods outside the FD region. In this study, we apply the FD calculations in a region encompassing the lowermost outer core and the topmost inner core. A constant grid spacing of 0.1 km by 0.1 km is used in the FD calculations. We use the solutions from the Global Centroid Moment Tensor (GCMT) project^36^ as focal mechanisms and the stacked PcP waveform as source time function.

Synthetic tests indicate that PKiKP waveform features have no sensitivity to the shear velocity structure in the top low-velocity layer, but some sensitivities to its density structure. However, density change alone is not sufficient to explain the waveform features. The maximum amplitude of the predicted secondary phase due to density change alone is no more than 35% of the main phase, while the amplitude of the secondary phase reaches 66% of the main phase in the observations. In the final model, we assume that shear velocity and density of the top low-velocity layer change in a same way as compressional velocity.

The double pulses observed in Fig. 3b cannot be fit by transitional ICB models, defined as that seismic medium parameters (compressional and shear wave velocities and density) increase linearly from the outer core values to the inner core values within a certain thickness *d* across the ICB (Supplementary Fig. 9a). Synthetic tests indicate that none of transitional ICB models, with the transitional thickness ranging from 1 to 10 km, could produce a secondary pulse in the synthetic waveforms and explain the observations (Supplementary Fig. 9b).

### Data availability

The data that support the findings of this study are available from the National Research Institute for Earth Science and Disaster Resilience (NIED), Japan, under its data policy. Data are also available from the authors upon request and with the permission of data redistribution granted by NIED.

## Electronic supplementary material

Supplementary Information
