# Supplementary material for: Seismological evidence for a localized mushy zone at the Earth’s inner core boundary
Source: Nat Commun. 2017 Aug 1;8:165. doi: 10.1038/s41467-017-00229-9 (PMC5537277; doi:10.1038/s41467-017-00229-9)
Supplement: Supplementary file 1 — Supplementary Information [file 41467_2017_229_MOESM1_ESM.pdf]

File name: Supplementary Information
Description: Supplementary Figure, Supplementary Tables and Supplementary References

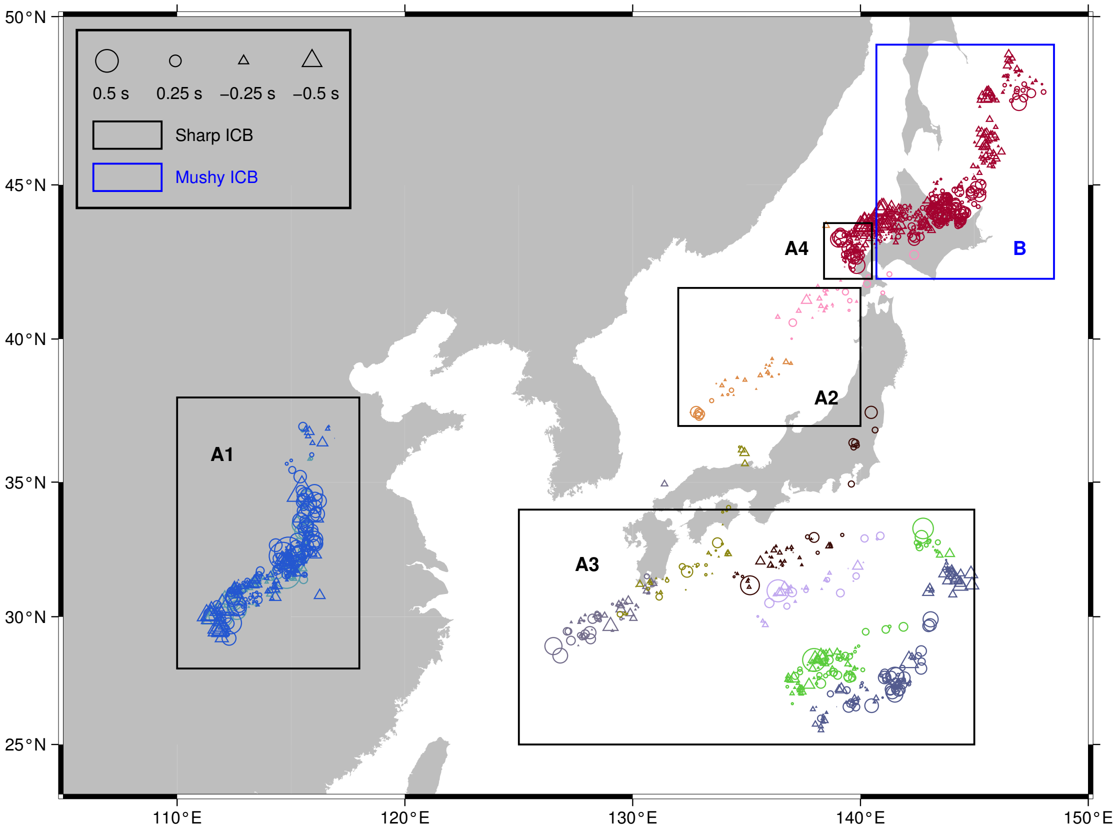

Supplementary Figure 1: Geographic distribution of PKiKP-PcP differential travel-time residuals. Residuals are plotted at the PKiKP reflected points at the ICB and color-coded with each event, with positive and negative values denoted by circles and triangles respectively, and the size of the symbol proportional to the absolute value of travel time residual. The ICB is grouped into regions according to their structural characteristics inferred based on seismic data, with black boxes (labeled as A: A1, A2, A3 and A4) indicating flat and sharp regions, and blue box (labeled as B) a region with a laterally varying double-layered structure (mushy zone) across the ICB. A cap-averaged version is shown in Fig. 2b.

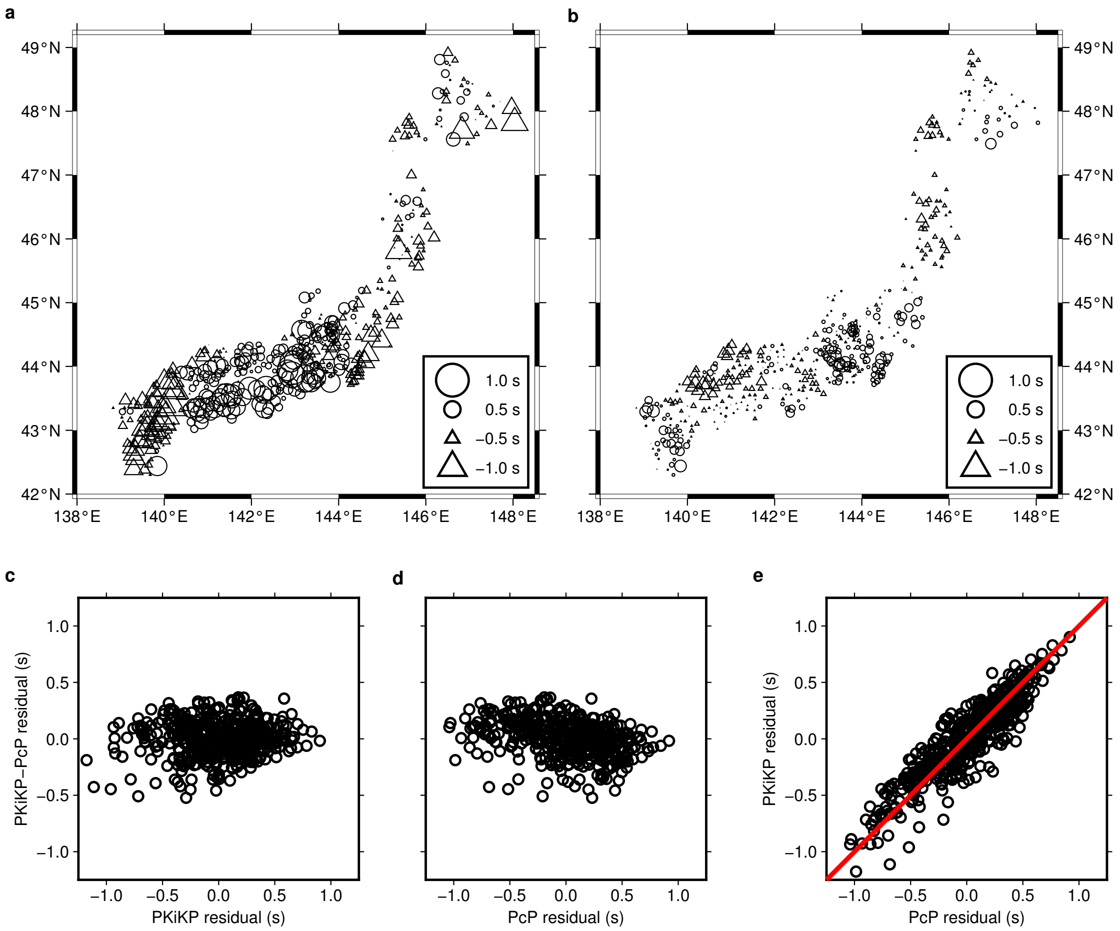

Supplementary Figure 2: Travel time residuals of event 11. a-b, Geographic distribution of **(a)** PKiKP travel time residuals and **(b)** PKiKP-PcP differential travel time residuals. All residuals are plotted at the PKiKP reflected points at the ICB, with positive and negative residuals denoted by circles and triangles respectively, and the size of the symbol proportional to the absolute value of travel time residual. **c-d**, Relationship between PKiKP-PcP differential travel time residuals and **(c)** PKiKP travel time residuals and **(d)** PcP travel time residuals. **e**, Relationship between PKiKP travel time residuals and PcP travel time residuals. Note that PKiKP travel time residuals exhibit a linear relationship with PcP travel time residuals.

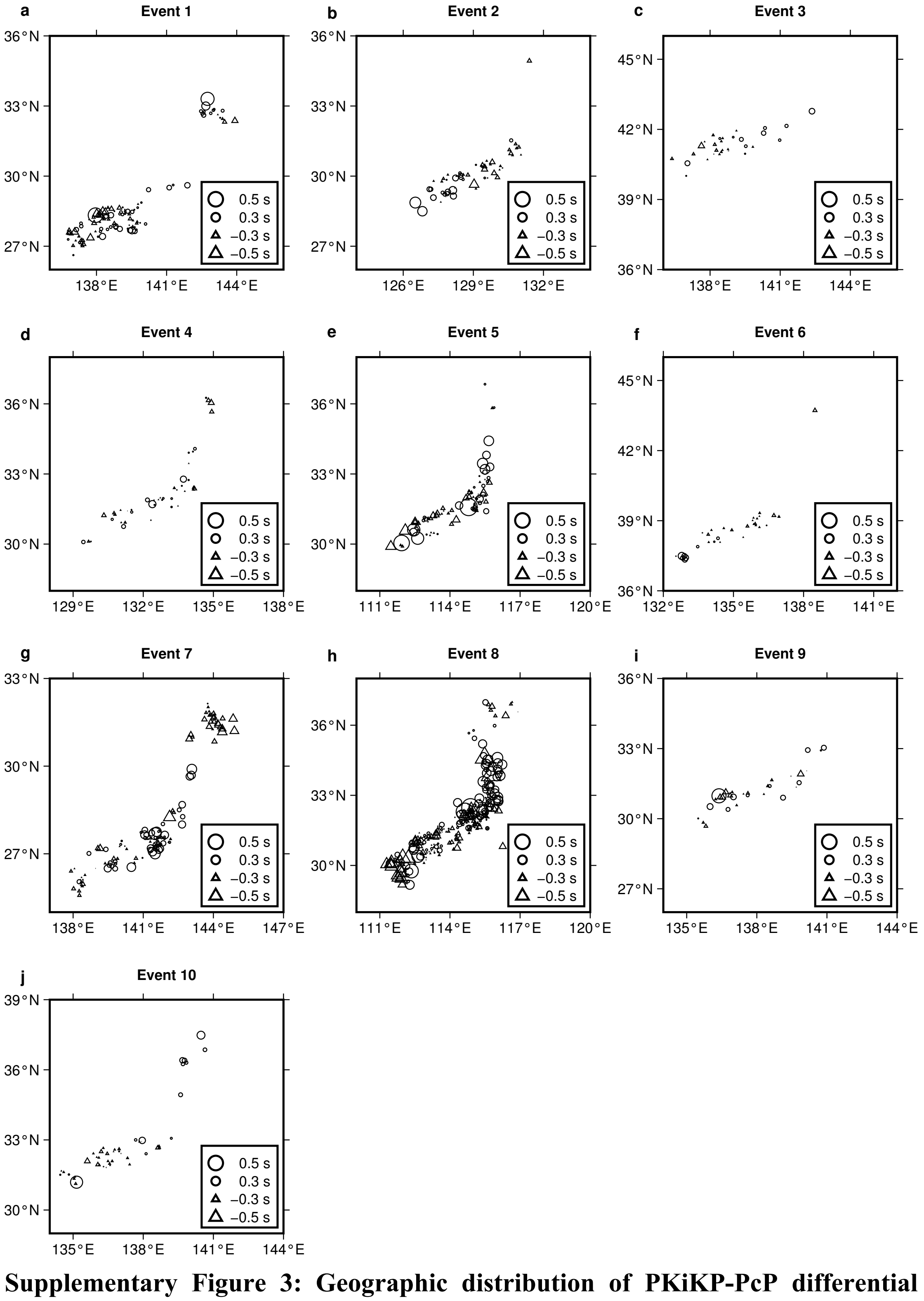

Supplementary Figure 3: Geographic distribution of PKiKP-PcP differential travel time residuals of all events except event 11. All residuals are plotted at the PKiKP reflected points at the ICB, with positive and negative residuals denoted by circles and triangles respectively, and the size of the symbol proportional to the absolute value of travel time residual.

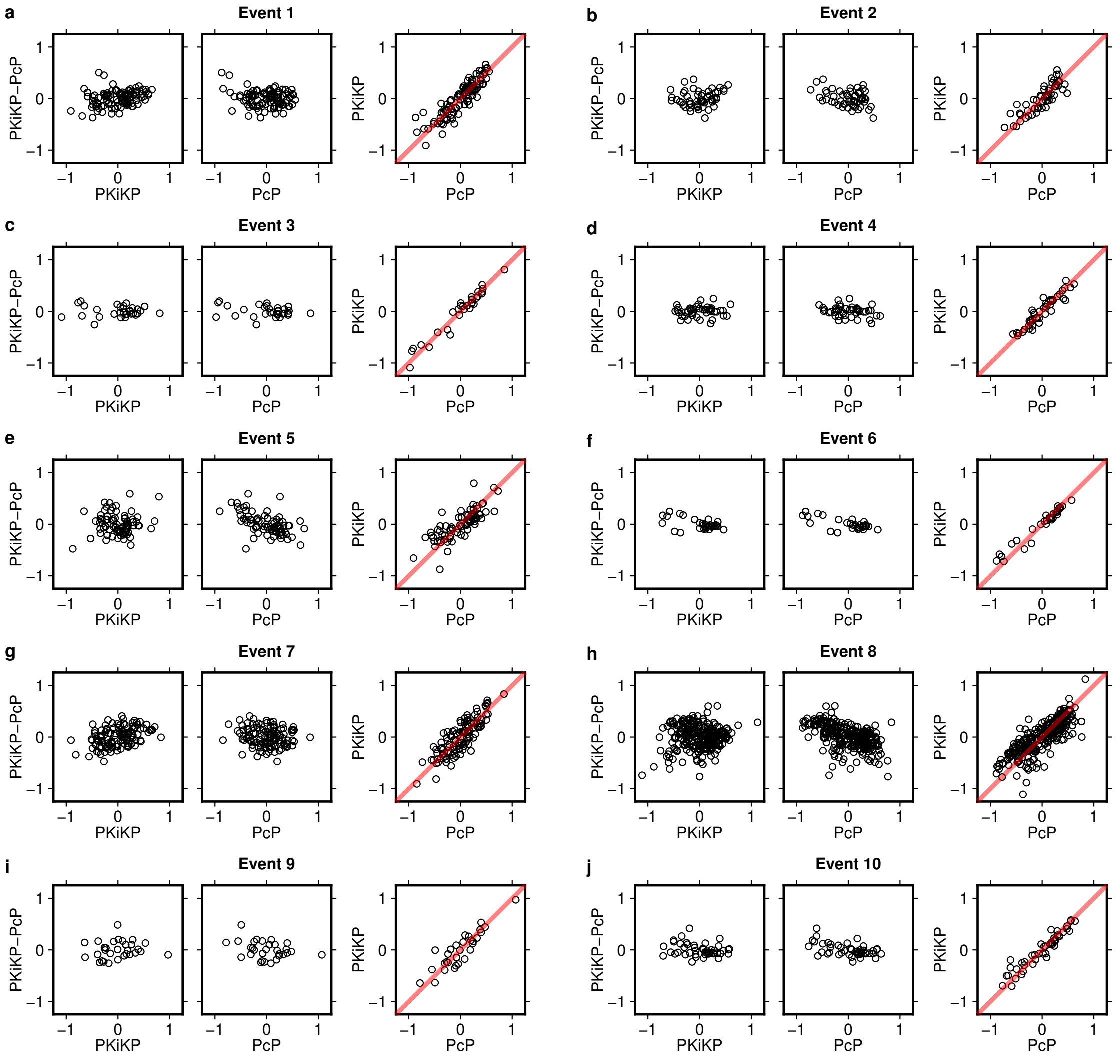

Supplementary Figure 4: Travel time residuals of all events except event 11. For each event, three relationships are presented: (left) relationship between PKiKP-PcP differential travel time residuals and PKiKP travel time residuals, (middle) relationship between PKiKP-PcP differential travel time residuals and PcP travel time residuals, and (right) relationship between PKiKP travel time residuals and PcP travel time residuals. Note that PKiKP travel time residuals exhibit a linear relationship with PcP travel time residuals.

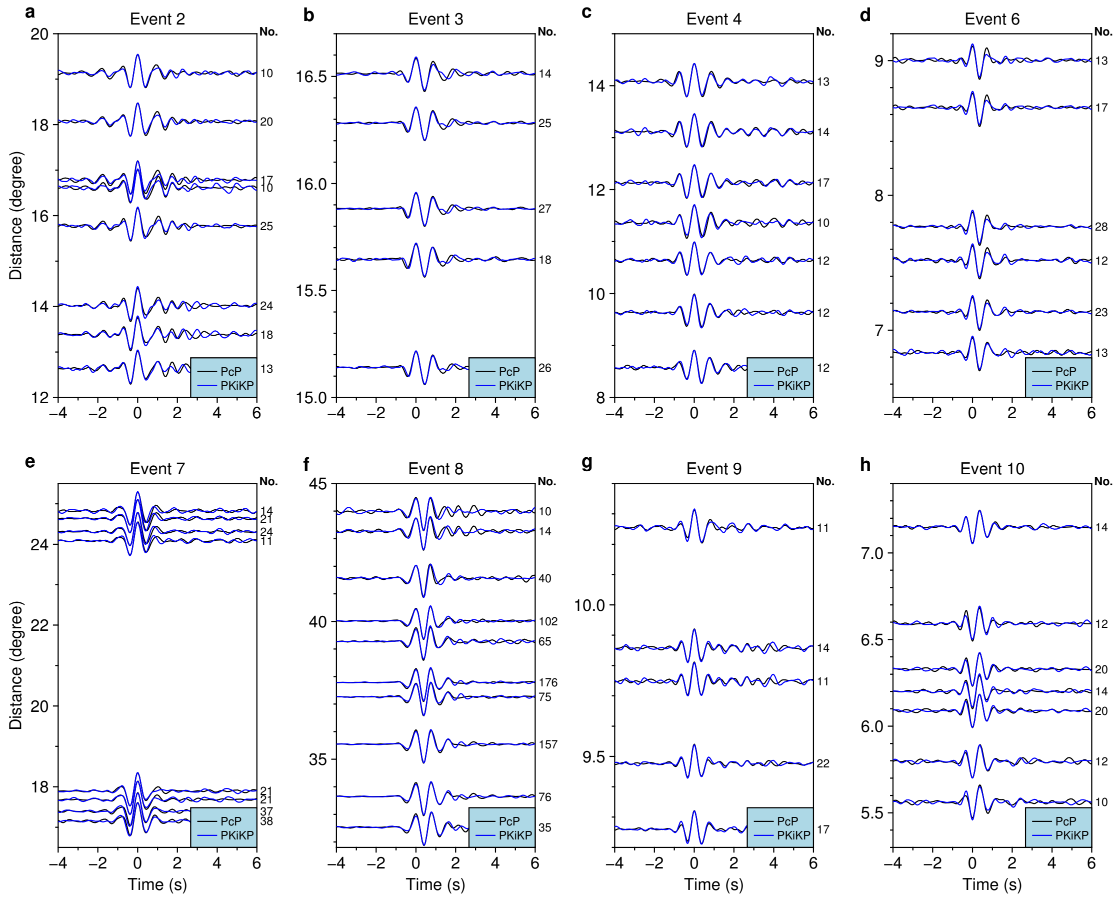

Supplementary Figure 5: Comparisons of stacked PKiKP and PcP waveforms in a frequency range of 1–3 Hz for all other events except events 1, 5 and 11. PKiKP and PcP waveforms are plotted in blue and black respectively. The number of waveforms used in each stacking is labeled at the right of each trace. Waveforms of event 1 are presented by Kawakatsu [1], and those of events 5 and 11 in Fig. 3.

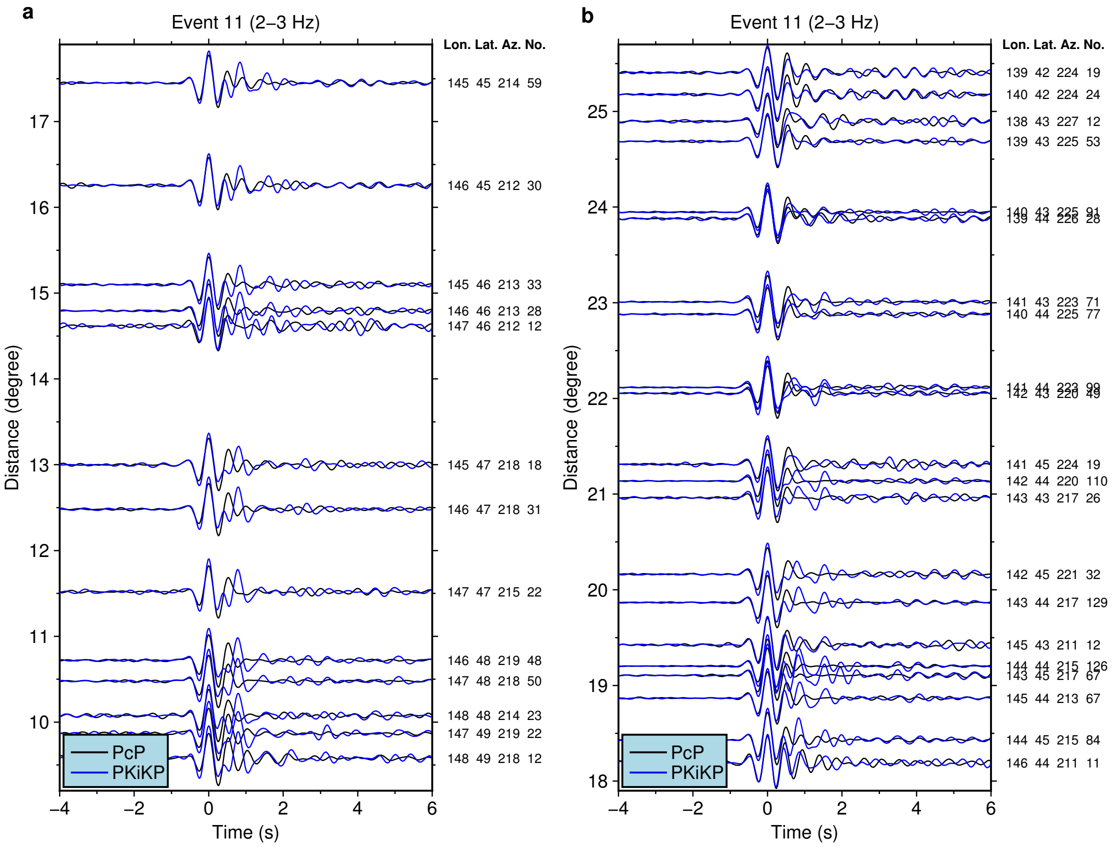

Supplementary Figure 6: Comparisons of stacked PKiKP and PcP waveforms of event 11 with a stacking radius *R*=1°. a-b, Stacked PKiKP (blue traces) and PcP (black traces) waveforms in the distance ranges of (**a**) 9°–18° and (**b**) 18°–26°. Geographical location of PKiKP sampling (longitude and latitude), azimuth of wave propagation direction, and number of waveforms used in each stacking are indicated at the right of each trace.

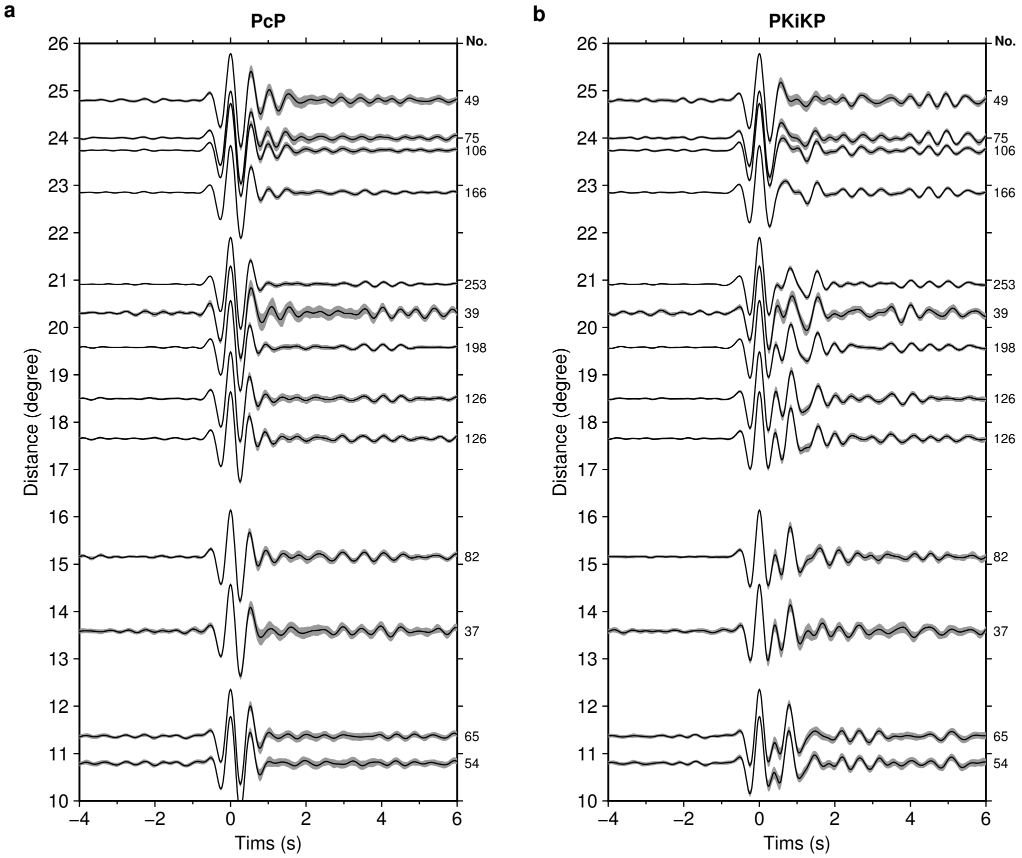

Supplementary Figure 7: **Uncertainties of stacked PcP and PKiKP waveforms. a**, Stacked PcP waveforms (black traces) and their 95% confidence intervals (gray shaded regions) obtained by a bootstrap resampling method[2]. The number of waveforms used in each stacking is labeled at the right of each trace. **b**, same as **a**, except for stacked PKiKP waveforms.

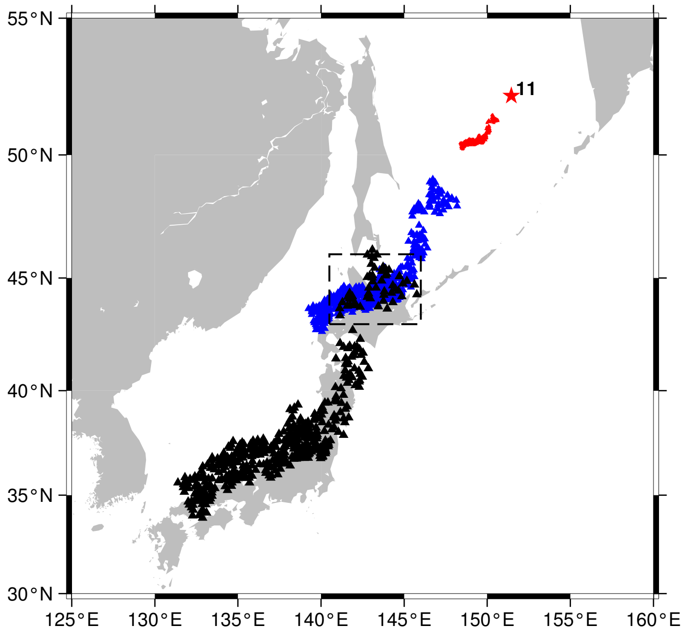

Supplementary Figure 8: Geographic locations of PcP reflected points, PKiKP entrant and exit points at the CMB of event 11. The location of event 11 is indicated by the red star. PcP reflected points, PKiKP entrant and exit points at the CMB are denoted by blue, red and black triangles, respectively. Note that the PKiKP exit regions partially overlap with the PcP reflected regions at the CMB (black dashed box).

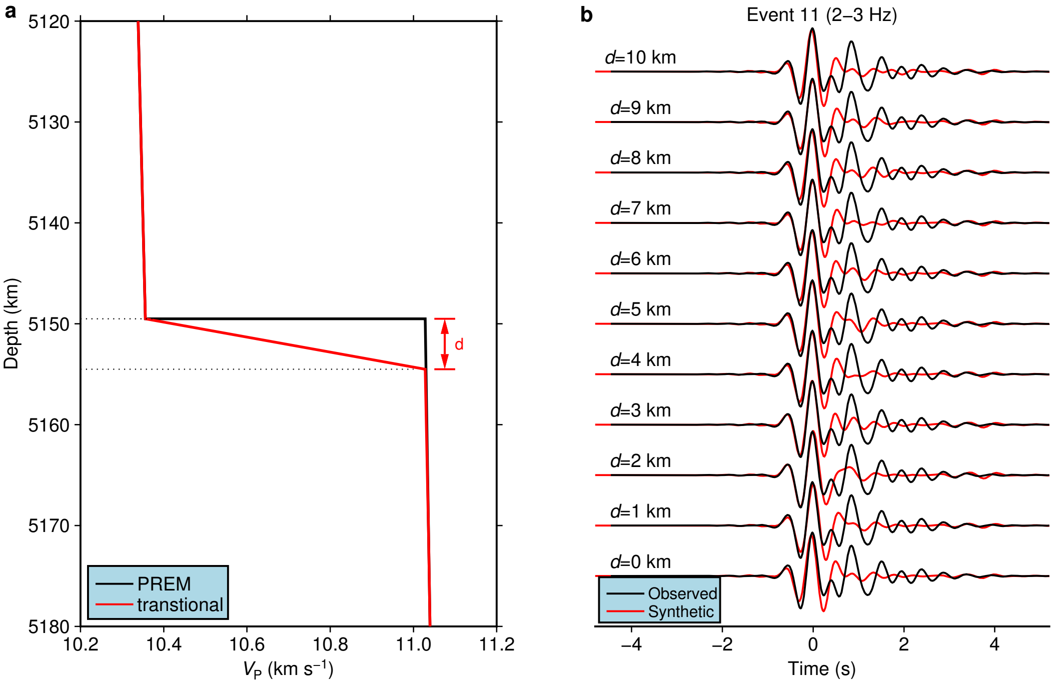

Supplementary Figure 9: Transitional ICB model and synthetics fitting to the PKiKP data. **a**, Compressional velocity profile of a transitional ICB model characterized by a transition thickness of *d* across the ICB (red line), along with PREM (black line). **b**, Comparisons of observed stacked PKiKP waveforms in a frequency range of 2–3 Hz for event 11 (black traces) and synthetic seismograms for various transitional ICB models (red traces), with the transition thickness labeled above each trace.

Supplementary Table 1: Event list used for PKiKP-PcP analysis.

Event ID	Origin Time (UTC)	Lat (°N)	Lon (°E)	Depth (km)	Mw	No. of Records
1	2001-07-03T13:10:46.280	21.635	142.988	325	6.5	106
2	2004-10-15T04:08:50.240	24.53	122.694	94	6.7	53
3	2004-11-07T02:02:26.170	47.948	144.477	474	6.2	31
4	2008-07-08T07:42:10.730	27.532	128.334	43	6.0	45
5	2009-09-03T19:51:07.629	24.355	94.702	104	5.9	80
6	2009-12-24T00:23:33.939	42.237	134.772	396	6.3	31
7	2010-03-08T09:47:08.890	19.348	144.743	427	6.1	116
8	2011-02-04T13:53:46.260	24.618	94.68	85	6.2	275
9	2012-05-26T21:48:10.119	26.91	140.055	487	6.0	30
10	2013-04-21T03:22:16.199	29.933	138.887	422	6.1	46
11	2013-05-24T14:56:31.460	52.235	151.444	624	6.7	450

Supplementary References

1 Kawakatsu, H. Sharp and seismically transparent inner core boundary region revealed by an entire network observation of near-vertical PKiKP. *Earth Planets Space* **58**, 855-863, (2006).

2 Efron, B. Bootstrap methods: another look at the jackknife. *Ann. Statist.* **7**, 1-26, (1979).
